# Effects of PCSK9 Inhibition on Coronary Atherosclerosis Regression of Nontarget Lesions after Primary Percutaneous Coronary Intervention in Acute Coronary Syndrome Patients

**DOI:** 10.1155/2022/4797529

**Published:** 2022-12-26

**Authors:** Yongjun Li, Mingming Yang, Xi Chen, Rui Zhang, Jing Li, Xiaoguo Zhang, Pengfei Zuo, Genshan Ma

**Affiliations:** ^1^Department of Cardiology, Zhongda Hospital, Medical School of Southeast University, Nanjing 210009, China; ^2^Department of Cardiology, Gaoyou Hospital of Traditional Chinese Medicine, Yangzhou 225600, China

## Abstract

**Aims:**

To evaluate the regression of coronary atherosclerosis with proprotein convertase subtilisin/kexin type 9 (PCSK9) inhibition in acute coronary syndrome (ACS) patients following primary percutaneous coronary intervention (PPCI). *Methods and Result*. We examined 40 nontarget lesions in 17 ACS patients who underwent PPCI and were treated with PCSK9 inhibitors. At 1 year, total cholesterol, low-density lipoprotein cholesterol (LDL-C), and atherogenic index (AI) decreased significantly by 2.5 mmol/L, 2.01 mmol/L, and 1.86, respectively. On quantitative coronary angiography, treatment with PCSK9 inhibitors reduced significantly the atherosclerotic area stenosis in nontarget lesions (61.18 ± 14.55 at baseline vs. 52.85 ± 15.51 at 1 year, *P* < 0.001).

**Conclusions:**

After 1 year of PCSK9 inhibition treatment for ACS patients, the area stenosis of non-TLR was considerably reduced.

## 1. Introduction

Hyperlipidemia is one of the most critical risk factors for coronary heart disease (CHD). According to estimates, there are up to 18.6 million CHD patients in the United States currently, among which acute myocardial infarction (AMI) is one of the most severe manifestations of CHD, with a high mortality rate of 14%, causing a grave threat to the patients' quality of life and survival [1]. Opening the criminal vessels as soon as possible is the best option for AMI, and primary percutaneous coronary intervention (PPCI) is currently the major treatment. However, some AMI patients who had PPCI are still at high risk of recurrent ischemic episodes, especially in the short term, which could lead to death in severe cases [2]. It has been shown that there is a three times higher cumulative incidence of nontarget lesion revascularization (non-TLR) than TLR in stented patients after three years of follow-up [3]. Moreover, patients with in-stent atherosclerosis have a considerably greater rate of non-TLR revascularization than those without it (78.6% vs. 44.4%, *P* = 0.028; another study with five-year follow-up) [4]. Even after successful PCI, non-TLR may contribute significantly to adverse cardiac events [5]. Hyperlipidemia is considered to play a crucial role in non-TLR [3]. Preventing effectively arteriosclerosis progression is of high importance for patients with coronary artery disease (CAD).

Clinical studies have shown that lowering plasma cholesterol levels, particularly low-density lipoprotein cholesterol (LDL-C), can delay the progression of atherosclerosis and play a significant role in reducing the incidence of major cardiovascular adverse events (MACE) in CHD patients [6, 7]. In addition, the ESC guidelines for the management of ST-segment elevation myocardial infarction (STEMI) recommend initiating lipid-lowering therapy as early as possible [8].

Evolocumab and alirocumab are novel developed proprotein convertase subtilisin/kexin type 9 (PCSK9) inhibitors that have been shown to be effective and safe in patients with stable angina and previous myocardial infarction [9–11]. PCSK9 inhibitors have been reported to have a rapid effect, a vast range of lipid reduction, a long duration of action, and an anti-inflammatory effect, providing a new dawn for further reducing MACEs of acute coronary syndrome (ACS) patients who underwent PCI [12–14].

In this study, quantitative coronary angiography was used to compare the area stenosis of nontarget lesions at baseline and 1 year after PCSK9 inhibition to determine the effect PCSK9 inhibition on coronary atherosclerosis regression of nontarget lesions after PPCI in ACS patients treated in combination with the standard statin therapy.

## 2. Methods

### 2.1. Study Population

In this study, 17 participants with ACS who underwent invasive PPCI between March 2019 and September 2020 were recruited. The subjects were injected subcutaneously with either evolocumab 140 mg every two weeks (or 420 mg monthly) or alirocumab 75 mg every two weeks (or 150 mg monthly) and were followed up for 1 year. Quantitative coronary angiography was employed to access the area stenosis of nontarget lesions at base line and after 1 year of treatment. This protocol was approved by the Institutional Ethics Committee for Clinical Research of Zhongda Hospital, Affiliated to Southeast University, and written informed consents were obtained from all participants.

### 2.2. Quantitative Coronary Angiography

All images were captured with the monoplane angiographic system (AXIOM Artis, Siemens, Erlangen, Germany) at 15 frames per second (fps) using routine angiographic projections. Necessary projection views were applied to acquire the best visualization of the lesions. The evaluation and quantitative analyses of angiograms were carried out by an experienced technician and a cardiologist. The area stenosis of the same lesion at the same angiographic projection angles before and after medication were then analyzed in the computerized angiographic workstation (Siemens, Erlangen, Germany) with normal adjacent segments as reference.

### 2.3. Statistical Analyses

Baseline characteristics and lipid levels at 1 year are expressed as means ± standard deviations (SDs) or medians with interquartile ranges for continuous variables and counts and percentages for categorical variables. Differences in lipid profile at the baseline and after 1 year were compared by using a Wilcoxon signed-rank test, and the changes in area stenosis of the lesions were evaluated using a paired Student's *t*-test. All the analyses were completed using Stata version 17 (Stata Corp, College Station, TX), with a conventional 2-tailed*P* < 0.05 considered as statistically significant.

## 3. Results

### 3.1. Baseline Characteristics

The baseline characteristics of 17 ACS patients are summarized in Table 1. 13 of them (76.47%) were males, and 52.94% had a history of smoking. BMI was averaged at 27.34 (range 25.39, 28.34). 52.94% and 17.65% of all patients had a history of hypertension and diabetes, respectively. The median triglyceride level was 1.61 (range 1.34, 2.32), while the median total cholesterol level was 4.95 (range 4.04, 5.36), the median HDL and LDL cholesterol levels were 1.13 (range 1.00, 1.29) and 2.81 (range 2.43, 3.50), respectively, and the median atherosclerosis index was 3.10 (range 2.84, 3.55) (see Table 1).

### 3.2. Clinical Impact of PCSK9 Inhibitors on the Changes in Lipid Profile and Coronary Atheroma of Nontarget Lesions

**Figure 1:**
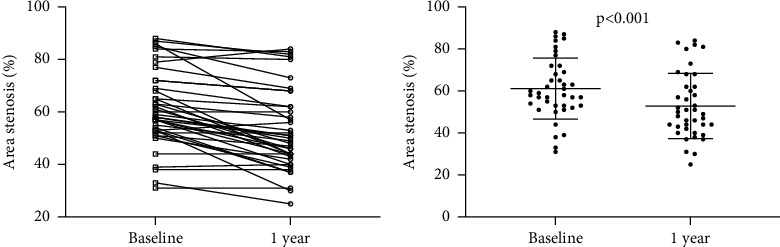
Changes in area stenosis from baseline to 1 year after treatment. (a) Changes in stenosis by patient; (b) mean changes in the stenosis.

As it is shown in Table 2, PCSK9 inhibitors dramatically lower total cholesterol, LDL cholesterol levels, and atherosclerosis index (*P* < 0.001). PCSK9 inhibitors considerably decreased triglyceride and HDL cholesterol, although this reduction was not statistically significant (*P* > 0.001). In general, PCSK9 inhibitors effectively optimize the lipid profiles of ACS patients. Importantly, PCSK9 inhibitors also significantly reduced the area of coronary atherosclerotic area stenosis in nontarget lesions (61.18 ± 14.55 at baseline vs. 52.85 ± 15.51 at 1 year, *P* < 0.001) (Figure 1).

## 4. Discussion and Conclusion

In this study, after discharge, all participants underwent angiography and a comparable course of treatment within 12 months of their discharge. At 1 year of follow-up, total cholesterol and LDL-C are much lower than at baseline, and this difference is statistically significant. After 1 year of the PCSK9 inhibitor therapy, the stenosis of nontarget lesions was also markedly reversed.

Currently, PCI is an effective treatment for CHD, especially for AMI. A considerable number of AMI patients have received timely medical treatment in recent years owing to the establishment and promotion of the National Chest Pain Center. However, some patients continue to experience negative clinical outcomes after PCI, and the incidence of these adverse events may negatively impact the clinical efficacy of PCI and even cause irreversible damage to the myocardium [2]. Previous studies have shown that the relative risk of major cardiovascular events and all-cause mortality decreases by 20% and 10% for each mmol/L reduction in LDL-C level, regardless of the levels of cardiovascular risk [15, 16]. Therefore, it is necessary to optimize the lipid-lowering strategy for these ACS patients who had PCI.

PCSK9, a member of the proprotein convertase family, is a serine protease that is mostly generated in the liver. By inhibiting LDL receptor (LDL-R) recirculation to the cell surface, PCSK9 upregulates LDL-C levels and improves LDL particle metabolism. PCSK9 is also detected in atherosclerotic plaques, contributing to the development and progression of atherosclerosis and thrombosis through a process involving platelet activation, leukocyte recruitment, as well as clot formation, and so on [17]. PCSK9 has been reported to regulate inflammation-related lipid metabolism in ACS, and its polymorphisms may be related to MACE [18]. PCSK9 is expressed in various cell types involved in the development and progression of atherosclerosis and can be detected in human atherosclerotic plaques [19].

Evolocumab, a kind of PCSK9 inhibitor, decreases LDL-C levels in plasma by inhibiting PCSK9-mediated LDL-R degradation and recycling LDL-R to the surface of hepatocytes. Accumulating evidence indicates that PCSK9 inhibitors are a safe, well-tolerated, and effective therapeutic strategy for patients with statin intolerance. In addition, LDL-C levels can be lowered to an unprecedented level with PCSK9 inhibition [20]. Alirocumab and evolocumab can lower LDL-C levels by up to 60% when added to standard statin therapy and simultaneously decrease the risk of recurrent atherosclerotic cardiovascular disease (ASCVD) events in ACS patients [21]. A systematic review and meta-analysis indicated that there is no statistically significant difference in common adverse events, serious events, and laboratory adverse events between PCSK9-mAbs treatment and placebo [22]. Importantly, the PCSK9-mAbs treatment significantly decreased LDL-C and other lipid levels while maintaining acceptable safety and tolerability [22]. In addition, a prior study has revealed elevated levels of PCSK9 in AMI patients [23], suggesting that reducing PCSK9 levels may provide additional benefits for AMI patients. According to the Fourier study, the combination of evolocumab and statins can considerably reduce LDL-C levels and MACEs in patients with high-risk cardiovascular disease, and there is no significant difference in safety between the treatment and control groups [9, 10]. In the GLAGOV study, 968 patients with symptomatic CHD were randomly assigned to receive evolocumab or placebo for 78 weeks on a statin basis, and the results showed that evolocumab could further lower LDL-C levels and induces regression of percent atheroma volume and total atheroma volume [24]. According to the findings of our study, treatment with PCSK9 inhibitors for 1 year considerably lowered TC and LDL-C levels, as well as the area stenosis of the lesions.

Lipid metabolism disorders play a key role in the formation of atherosclerotic plaque. PCSK9 can effectively prevent the formation of plaque by lowering TC and LDL-C levels and improving lipid metabolism. Although TC and LDL levels were much lower than before, plaque area in some individuals grew or remained unchanged. That could be because the formation of atherosclerotic plaque is not only related to lipid deposition on the vascular wall but also to inflammatory response. It is widely acknowledged that chronic inflammation plays an important role in both the onset and progression of atherosclerosis [25]. However, other risk factors, including hypertension, diabetes, and smoking, can also cause endothelial cell injury and inflammation. Therefore, a nonsignificant change or even increased plaque area of individual patients after medication may not be excluded as a cause of poor control of these high-risk factors.

We are quite aware of several limitations of our study. First of all, we only recruited 17 ACS patients. Second, we did not compare the effects of PCSK9 inhibitors in patients with those on standard statin therapy. Finally, we need to examine a wider profile of patients' characteristics before and posttreatment in a larger cohort.

In conclusion, PCSK9 inhibitors used in combination with standard statin therapy can further lower TC and LDL-C levels, thereby promoting the regression of plaque and reducing the risk of adverse cardiovascular events caused by atherosclerosis progression.

## Figures and Tables

**Table 1:** Baseline characteristics of the patients (*n* = 17).

Variables	Mean ± SD/median (Q1-Q3)/*n*(%)
Age (years)	52.35 ± 12.82
Male	13 (76.47)
Smoking	9 (52.94)
BMI (kg/m^2^)	27.34 (25.39, 28.34)
Hypertension	9 (52.94)
Diabetes mellitus	3 (17.65)
Triglycerides (mmol/L)	1.61 (1.34, 2.32)
Total cholesterol (mmol/L)	4.95 (4.04, 5.36)
HDL cholesterol (mmol/L)	1.13 (1.00, 1.29)
LDL cholesterol (mmol/L)	2.81 (2.43, 3.50)
AI	3.10 (2.84, 3.55)

BMI, body mass index; HDL, high-density lipoprotein; LDL, low-density lipoprotein; AI, atherogenic index = (TC − HDL)/HDL.

**Table 2:** Changes in the lipids profile.

	1-year median (Q1-Q3)	Change from the baseline mean (95% CI)	*P* value ^*∗*^
Triglycerides (mmol/L)	1.09 (0.80, 1.57)	−0.77 (−1.56, 0.22)	*P* = 0.0312
Total cholesterol (mmol/L)	2.26 (2.11, 2.45)	−2.50 (−2.98, −2.02)	*P* = 0.0003
HDL cholesterol (mmol/L)	1.06 (0.94, 1.11)	−0.13 (−0.23, 0.03)	*P* = 0.0191
LDL cholesterol (mmol/L)	0.95 (0.85, 1.16)	−2.01 (−2.46, −1.57)	*P* = 0.0003
AI	1.31 (0.90, 1.43)	−1.86 (−2.17, −1.56)	*P* = 0.0003

^*∗*^Compared with baseline using Wilcoxon signed-rank test.

## Data Availability

The data used to support the findings of this study are available from Yongjun Li (e-mail: liyongjunnj@hotmail.com) upon request and with permission by Zhongda Hospital.
